# Novel non-invasive algorithm to identify the origins of re-entry and ectopic foci in the atria from 64-lead ECGs: A computational study

**DOI:** 10.1371/journal.pcbi.1005270

**Published:** 2017-03-02

**Authors:** Erick A. Perez Alday, Michael A. Colman, Philip Langley, Henggui Zhang

**Affiliations:** 1 Biological Physics Group, Department of Physics and Astronomy, University of Manchester, Manchester, United Kingdom; 2 Theoretical Physics Division, Department of Physics and Astronomy, University of Manchester, Manchester, United Kingdom; 3 School of Biomedical Sciences, Faculty of Biological Sciences, University of Leeds, United Kingdom; 4 School of Engineering, University of Hull, Hull, United Kingdom; Universiteit Gent, BELGIUM

## Abstract

Atrial tachy-arrhytmias, such as atrial fibrillation (AF), are characterised by irregular electrical activity in the atria, generally associated with erratic excitation underlain by re-entrant scroll waves, fibrillatory conduction of multiple wavelets or rapid focal activity. Epidemiological studies have shown an increase in AF prevalence in the developed world associated with an ageing society, highlighting the need for effective treatment options. Catheter ablation therapy, commonly used in the treatment of AF, requires spatial information on atrial electrical excitation. The standard 12-lead electrocardiogram (ECG) provides a method for non-invasive identification of the presence of arrhythmia, due to irregularity in the ECG signal associated with atrial activation compared to sinus rhythm, but has limitations in providing specific spatial information. There is therefore a pressing need to develop novel methods to identify and locate the origin of arrhythmic excitation. Invasive methods provide direct information on atrial activity, but may induce clinical complications. Non-invasive methods avoid such complications, but their development presents a greater challenge due to the non-direct nature of monitoring. Algorithms based on the ECG signals in multiple leads (e.g. a 64-lead vest) may provide a viable approach. In this study, we used a biophysically detailed model of the human atria and torso to investigate the correlation between the morphology of the ECG signals from a 64-lead vest and the location of the origin of rapid atrial excitation arising from rapid focal activity and/or re-entrant scroll waves. A focus-location algorithm was then constructed from this correlation. The algorithm had success rates of 93% and 76% for correctly identifying the origin of focal and re-entrant excitation with a spatial resolution of 40 mm, respectively. The general approach allows its application to any multi-lead ECG system. This represents a significant extension to our previously developed algorithms to predict the AF origins in association with focal activities.

## Author summary

Atrial tachy-arrhythmias are associated with irregular excitation waves arising from re-entrant excitation, multiple wavelets or rapid focal activity. Identifying the origin of the irregular activity may be vital for diagnosis and treatment of the disorder. Where invasive and non-invasive methods provide approaches for such identification, both have associated disadvantages. In this study, we used a biophysically detailed model of the human atria and torso to develop an algorithm based on the correlation between the electrocardiogram (ECG) signal from a 64-lead vest and the location of rapid focal and re-entrant excitation. Using the properties of the atrial activation and the ECG signals, we developed a focus-location algorithm which is able to distinguish rapid focal activity from re-entrant scroll waves centred in the same location. Based on simulated data, the algorithm had success rates of 93% and 76% for correctly identifying the origin of focal and re-entrant excitation, respectively, and 88% for distinguish focal and re-entrant excitation, with no false positives. Inherited from our previous algorithm, it is also easily generalizable to any multi-lead ECG system.

## Introduction

Atrial tachy-arrhythmias, including atrial fibrillation (AF), atrial tachycardia (AT) and flutter (AFL), are the most common cardiac arrhythmias, predisposing to heart attack, stroke and even possible cardiac death [1,2]. All three are characterised by rapid and irregular electrical activation of the atria, with AF presenting the greatest complexity. Such rapid and irregular electrical activity of the atria is normally associated with one or more of the following abnormal excitation patterns: focal pacing (spontaneous rapid firing of non-pacemaker cells) [3,4], fibrillatory conduction of multiple wavelets [5] and re-entrant excitation scroll waves (i.e., rotors) [4,5].

Epidemiological studies have shown an increase in AF prevalence in the developed world associated with an ageing society, highlighting the need for effective treatment options [6,7]. Current treatment of AF involves the use of rate control, anticoagulation, cardioversion and ablation [8]. The restoration of sinus rhythm in the atria may improve cardiac function, however several drug treatments have limited efficacy in long term maintenance of sinus rhythm [6,9]. Developments aiming to reduce the critical mass required to sustain AF, such as catheter-based radio-frequency ablation therapy, have proven to be more effective in suppressing AF substantially [9], although multiple procedures may still be necessary due to high recurrence rates [10].

For a successful AF ablation, it is vital to know the origins (i.e., the driving sources) of AF prior to the procedure, because isolating the driving source from the rest of the atria is the primary goal of such therapy [9]. To identify such origins, both invasive and non-invasive techniques have been developed. These include the low density endo-surface mapping technique of 64-electrode basket catheters [11] and electrocardiogram imaging (ECGi) [12]. The main limitation of using an invasive method is that it might produce further complications during the surgery [13]. There is a pressing need to develop effective non-invasive methods to identify AF origins which might provide all of the necessary information prior to the surgery. The ECGi technology, based on the inverse problem solution [12], is a promising method in clinical diagnosis. However, current algorithms require further information to constrain the solution to achieve a reliable reconstruction of cardiac excitation waves due to the ill-posedness of the problem [14].

Recent studies have also developed algorithms to identify non-invasively the location of focal sources by using either the standard 12-lead [15–17] or multiple-lead (e.g. 64-lead) ECG systems [18,19]. The success rates of these algorithms range from 40 to 90%. Most are based on the correlation between the location of focal activity and the P-wave morphology or polarity [16–18]. Whereas they are useful in identifying the origin of focal excitation, current algorithms may not be applicable to identify re-entry or very rapid focal activity; at such rapid rates, regular and irregular fragmented waves are typically observed and thus determination of morphology or polarity of the main activation wave is non-trivial. Confounding the case for re-entry, atrial flutter waves or F-waves are also likely to be more fragmented and more complex in nature. It is also important to be able to distinguish very rapid focal activity from that of re-entry at a comparable rate, as the underlying maintenance mechanisms in these conditions are different and thus it is possible that different intervention may be required to terminate the arrhythmia.

The aim of this study is to go-beyond our previous studies [17–19] in identifying the origins of focal-related AF from body surface ECG to develop a novel algorithm based on rapid regular atrial waves in order to identify origins for both rapid focal and re-entrant activity from a multi-lead ECG system.

## Methods

### Atrial-torso model

**Fig 1:**
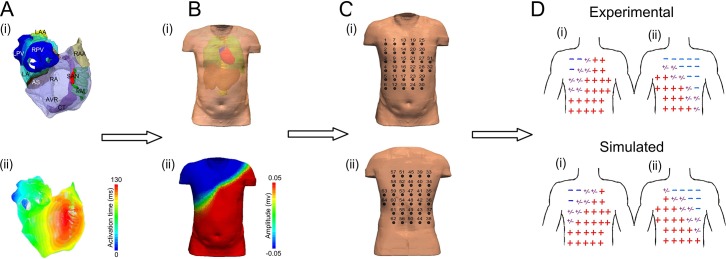
Models and procedure used to develop the algorithm. Illustration of atria (A) and torso (B) models used in the study to simulate re-entry and ectopic activity in the atria. (C) Electrode positions used to simulate the 64-lead ECG. (D) Simulated anterior (i) and posterior (ii) polarity map, as compared to experimental data, validating the 3D atria-torso models.

A previously validated biophysically detailed computational model of the three-dimensional (3D) human atria and torso [17,18,20] was used to simulate ectopic focal and re-entry conditions (Fig 1). The atrial model was segmented into the major anatomical structures and accounts for electrophysiological heterogeneity between these regions (Fig 1A) [21]. The model has been previously used and determined suitable for studying atrial arrhythmia mechanisms [17,22]. The atrial model was placed into a previously developed and validated male torso model which accounts for the segmented structure of lungs, liver, blood masses and spinal cord and the respective electrical conductivities (Fig 1B) [18,20]. A table of conductivity values used can be found in supplementary information (S1 Table). For further test, a previously developed and validated female torso geometry [18] was implemented (S1 Text). Both models have been used before to develop an algorithm to diagnose atrial ectopic origin from multi lead ECG systems [18]. Details of the atrial cell models and 3D simulation protocols can be found in Colman *et al* [22] or in S2 Text and single cell code S3 Text; details of the torso model development, validation and simulation protocols can be found in Perez Alday *et al* [18].

### Simulating atrial rapid ectopic foci and re-entry

**Fig 2:**
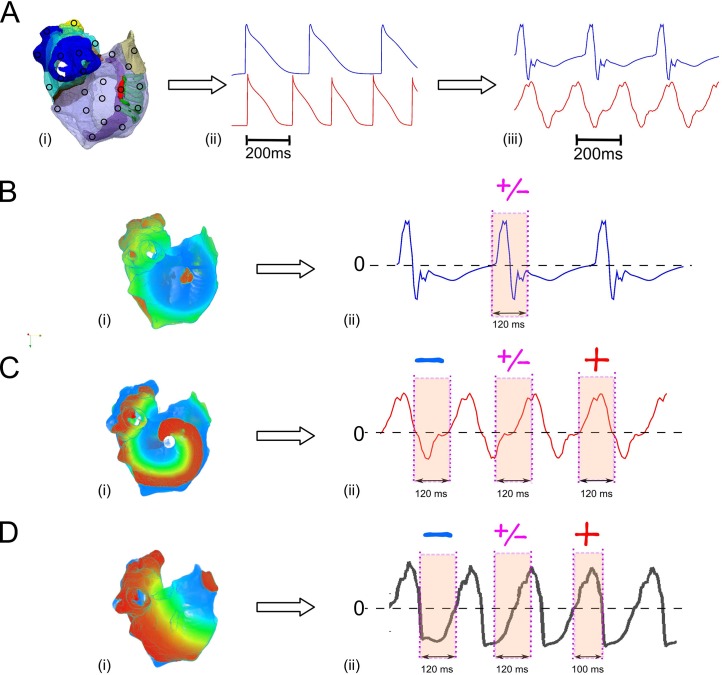
Illustration of different atrial activation associated with different body surface atrial waveform morphology. (A) Different stimulated points (circle) and tip of re-entry across the surface of the atria (i), atrial action potentials (ii) and their corresponding body surface atrial-waves at different excitation rates (iii): top 3Hz; bottom 5Hz. (B) Snapshot of atrial activation at control conditions at a fast rate, (i) and its corresponding ECG exhibiting distinct P-wave in lead V1 (ii). (C) Snapshot of atrial activation when the tip of the re-entry is located in the SAN (i), and its corresponding atrial waves of lead V1 (ii). (D) Snapshot of atrial activation with the focal ectopic activity located in the right atrial appendage (RAA) (i) and the corresponding atrial waves of lead V1 (ii). A red sign represents a positive polarity in the atrial-wave (magenta/shaded area), the blue sign is a negative polarity and a purple sign represents a biphasic atrial-wave (magenta/shaded area).

Ectopic focal and re-entrant excitations were initiated in different regions of the atria (Fig 2Ai). 2D views and 3D vtk files can be found in supplementary information S. In order to allow rapid excitation waves with rates at frequencies typical of AF/AT/AFL (i.e. 2.5–8 HZ [22,23]) to be sustained in the atria, parameters of the Colman *et al*. model of single human atrial myocytes were modified to incorporate experimentally observed AF-induced electrical remodelling of ion channels [22], which resulted in shortened AP (Fig 2A). To simulate ectopic focal activity, a sequence of external supra-threshold electrical pulses (with amplitude of 2nA and duration of 2-3ms) was applied to various locations across different regions of the atria (Fig 2Ai). Re-entrant excitation waves were initiated by a phase distribution method [24,25]. Although this is an artificial method for initiating re-entrant excitation, it allows the location of the centre of the rotor wave to be easily controlled. To avoid possible effects of the transition period of excitation waves on their kinetics due to the unphysiological initiation procedure, data after 1 second of initiation were analysed. In cases where re-entrant scroll waves were not localised to the initiation point, i.e. there was a degree of meander, a small non-excitation area (0.5 cm in radius) was incorporated around a specific region of the atria, in order to stabilise the rotor centre (Fig 2C). This allowed sustained re-entrant activity with its origin (i.e. tip) located in a specific region of the atria to be produced. The inclusion of a small area of non-excitable tissue did not produce a marked change in tissue’s volume or morphology of the measured potential on the body surface. In simulations, cases when re-entrant excitation waves had a significant degree of meander were used to test the ability of the algorithm to track the tip of the scroll waves spatio-temporally.

To test the algorithm’s ability to distinguish between focal and re-entrant activities centred on the same spatial locations, a set of focal stimuli simulations were matched in location and excitation rate to re-entrant simulations centre at multiple locations.

### Simulating body surface potential

A boundary element method (BEM) was used to calculate the potential on the surface of the torso [26]. From the body surface potential (BSP), 64-lead ECG signals were obtained by selecting elements of the torso mesh corresponding to the position of the electrodes as described in previous studies [18,20]. The P-wave of the 64-lead ECG during control conditions matched the experimental data of multiple patients [18,27] (see S1 Fig), validating the development of the heart-torso model.

### Measurement of potentials of positive and negative poles

From the measured atrial-waves, the dynamical evolution of the spatial distribution and amplitude of the atrial-wave dipole was computed from the 64-lead ECG, following the same method as used in previous experimental studies [18,27]. The dipole pattern on the body surface was reconstructed by selecting the maximum positive potential value (positive pole) and the minimum negative potential value (negative pole) of the 64-lead ECG at every time step [27]. The amplitude and the spatial pattern of the atrial-wave dipole based on the 64-lead ECG changed with time as the atrial activation evolved. In the model, both the amplitude and the temporal evolution of the dipole location agreed with the experimental data [18] during control conditions (S2 Fig), further validating the model development.

### Algorithm to locate the atrial source

In a previous study, we developed an algorithm to identify the location of atrial ectopic focal activity, using the polarity map on the body surface potential that was produced from a 64-lead ECG system, which was split into two sets of quadrants (anterior/posterior) [18]. The algorithm was based on the fact that a negative polarity P-wave in a certain lead implied an excitation wave propagating away from the positive electrode of that lead. Thus, the quadrant of the 64-lead electrode positions with the largest number of electrodes with negative P-waves would correlate directly to the origin of the focal excitation. The success rate of the algorithm was 93%, meaning that it correctly identified the origin of atrial focus in 75/80 simulations.

However, the previous algorithm was only able to detect slow ectopic focal atrial activation from body surface potential mapping and cannot be applied directly to detect the origin of atrial excitation waves due to rapid focal or re-entrant activity because of the complexity of the body surface waveform, which produces fibrillatory waves. Determining the polarity of fast atrial waves is not trivial since it may consist of positive, negative and biphasic waves, depending on the time period investigated (Fig 2C(ii) and 2D(ii)). Furthermore, re-entrant and focal excitation patterns may present different characteristics of fragmented fast atrial waves, and the ability to distinguish between these types of excitation could provide valuable information for directing treatment. Thus, in order to apply our previously developed algorithm to both rapid focal and re-entrant excitation, new tools were developed. The first tool was to determine the polarity of the atrial wave associated with main atrial activation, in order to identify the location of the source. The second tool was to quantify the differences between focal and re-entrant activity. Further details of these algorithmic developments are provided below.

### Determining the polarity of atrial waves

At slow pacing rates, it is straightforward to determine the polarity of individual P-waves: the long period of the diastolic phase means that the ECG signal remains at a baseline during this interval, with a clear deflection from the baseline corresponding to atrial activation during the systolic period (Fig 2B). This deflection is the P-wave, and may be positive, negative or biphasic (with both positive and negative portions). The duration (i.e., the time interval) of the P-wave corresponds to the time interval of atrial activation.

The challenge for determining the polarity of the atrial wave at rapid pacing rates is that the diastolic period is absent, leaving the ECG signal absent of a stable baseline. Therefore, there is no clear distinction between successive deflections (Fig 2C(ii) and 2D(ii)). Determination of the polarity of the atrial wave in such case is thus non-trivial; any polarity can be extracted from the same signal, depending on the time interval which is considered (Fig 2C(ii) and 2D(ii)). However, the polarity in the interval during which a large volume of the atrial mass is excited (i.e. main atrial activation) can be determined and is suitable for our algorithm. Thus the time interval corresponding to the main atrial activation must first be determined.

**Fig 3:**
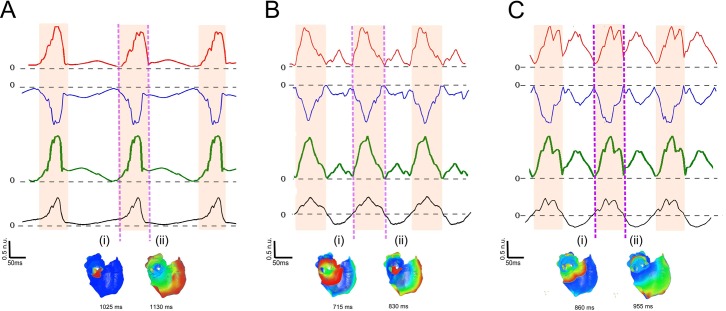
Dipole and atrial activation evolution in different atrial activations located in the pulmonary veins (PV). (A) Slow ectopic atrial activation focus in the PV (non fibrillatory waves observed). (B) Fast focal activation focus in the PV (F-waves observed). (C) Re-entrant activation around the PV (F-waves observed). Red line: Positive dipole. Blue line: Negative dipole. Green line: Dipole sum. Black line: lead V1 (ECG). The Magenta regions represent the time interval of the main atrial wave, selected from the peaks in the dipole pattern. (i)-(ii) Snapshots of the atria activation at the beginning and end of the time interval selected.

Analysis shows that the dipole signal provides sufficient information to determine the time interval of main atrial excitation (Fig 3). Fig 3 illustrates results for three different cases of atrial activation originating from the same location but with increasing complexity (i.e. slow focal pacing, rapid focal pacing, and re-entrant excitation).

At the slow rate, determination of the polarity of the P-wave is straightforward and can be seen to be positive in lead V1 (Fig 3A–black line). Note that the time interval of the P-wave indeed corresponds to the time interval of the atrial activation (Fig 3A(i) (ii)). Also, both positive and negative poles of the body surface dipole have one significant deflection, and the time interval of this deflection corresponds directly to the time interval of atrial activation and therefore the P-wave (Fig 3 –red and blue lines). The positive and negative dipole signals can be combined as a “dipole sum” (defined as the sum of the modulus of the negative and positive poles), giving a single signal with a significant deflection corresponding to the time interval of atrial activation (Fig 3 –green line).

At rapid rates where F-waves rather than P-waves are observed, there are no clear markers for the time interval of atrial activation in the ECG signal (Fig 3B and 3C–black line). The dipole sum, however, still presents a signal with one easily identifiable prominent deflection; the time interval of this deflection corresponds to the main atrial activation (Fig 3B(i),(ii)), even in the case of more fragmented atrial waves resulting from re-entrant activity (Fig 3C(i),(ii)). The portion of the atrial wave within this time interval therefore gives the polarity associated with the main atrial activation. In examples shown in Fig 3, the polarity is positive in lead V1 for all cases but the polarity will vary spatially across the body surface according to lead position.

Thus, by selecting the ECG segment corresponding to the main atrial activation (obtained from dipole sum, Fig 3 magenta/shaded regions), the polarity (positive, negative or biphasic) of each lead in this segment is determined.

### Atrial source location based on the atrial-wave polarity map

Having identified the polarity of the fast atrial waves in each lead, the resulting 64-lead polarity distribution feeds directly into our original atrial source location algorithm [18].

**Fig 4:**
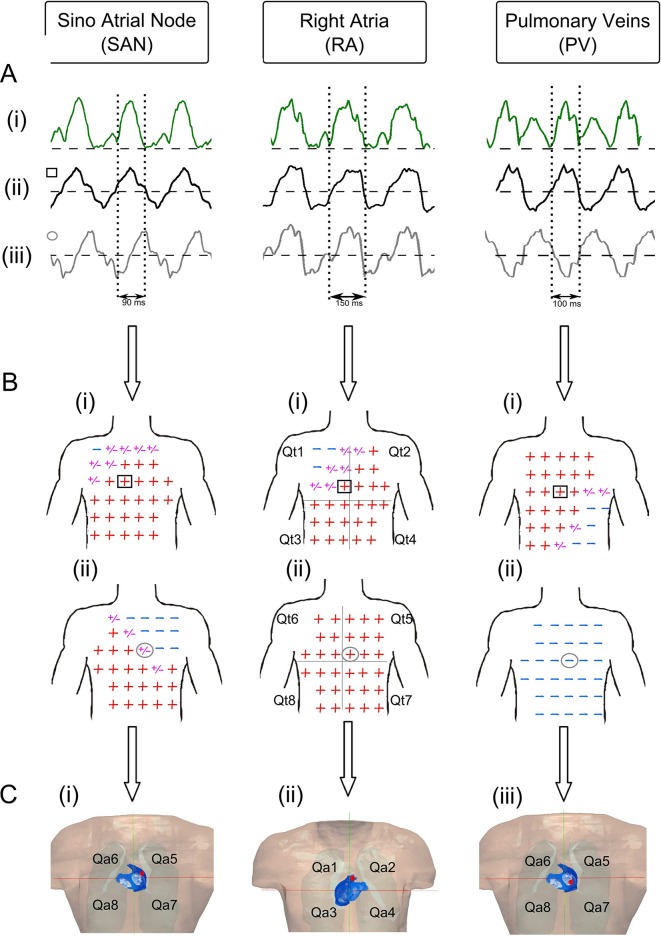
Illustration of the correlation used by the algorithm for activation from three atrial sites. (A): Dipole sum (green line) (i), lead V1 (black line) (ii) and Lead 47 (Grey line)(iii), (i) were used to identify the time interval (section between dotted lines) of re-entrant patterns where the tip was located in the sino-atrial node (SAN), right atria (RA) and pulmonary veins (PV). The amplitude in all cases has been normalized. (B): Atrial-wave polarity map in the anterior (i) and posterior (ii) part of the torso for atrial activation initiated at different locations of the atria (SAN, RA and PV). A red sign represents a positive polarity in the atrial-wave, the blue sign is a negative polarity and a purple sign represents a biphasic atrial-wave. The black square represents the electrode position of lead V1, and the grey circle represents the electrode position of lead 47. (C): Rotor tip (red dot) identified by the algorithm in each simulation. The anterior (ii) and posterior (i) parts of the atria and torso (B-i,ii) are shown for each case. In each case the algorithm correctly identifies the correct quadrant: SAN the tip is located in the quadrant Qa5 (i), for RA the tip is located in the quadrant Qa2 (ii), and for PV the tip is located in the quadrant Qa7 (iii).

Fig 4 demonstrate the correlation used by the algorithm to determine the origin of non-meandering atrial re-entrant activations, centred on the sino-atrial node (SAN) (left), right atrial appendage (RAA) (middle) and pulmonary veins (PV) (right). In each case, the time interval has been obtained by selecting the largest deflection in the dipole sum evolution pattern (Fig 4A- vertical dashed lines) as described in the previous section. Then, an atrial-wave polarity map is created (Fig 4B) from the time interval selection. Once the polarity map has been created (Fig 4A and 4B), the location of the source of the atrial activation can be found through the Perez Alday *et al*. algorithm [18] (S3 Fig), which associates the two set of torso quadrants (Qti) (Fig 4B) with the two set of atria quadrants (Qai) (Fig 4C (i)-(ii)).

### Differentiating ectopic focal from the re-entrant activity

**Fig 5:**
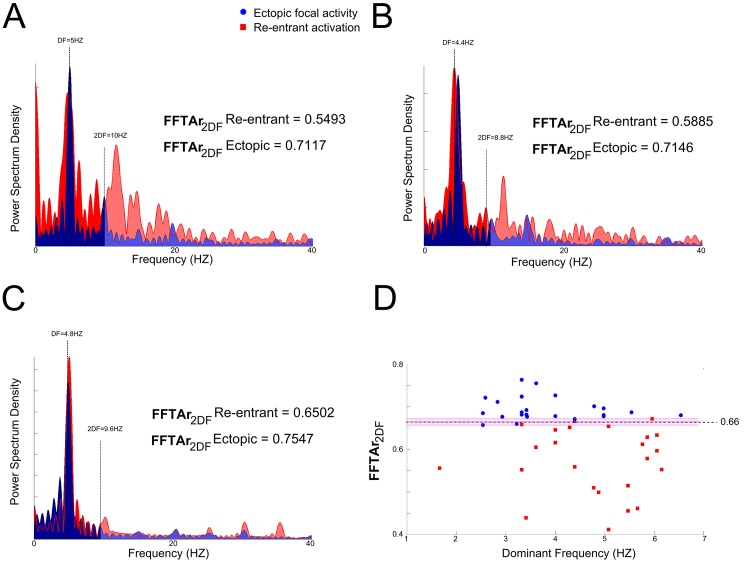
Normalized power spectral density for ectopic focal and re-entrant activation. Normalized power spectral density for ectopic focal (blue) and re-entrant (red) activity located in (A) SAN; (B) PV and (C) RAA. The darker shadow corresponds to the area between 0–2 x DF. (D): a scatter plot of (AFFTr_2DF_) against the DF, the magenta area is the overlapping area where both activities can occur. AFFTr_2DF_ is the ratio of the area under the power spectrum density in the ranges 0 –(2 x DF) Hz and (2 x DF)– 50 Hz: AFFTr_2DF_ = Area_0-2DF_/Area_0-50Hz_

Our simulations demonstrate that re-entrant excitation waves are characterised by more fragmented ECGs (Fig 3C) compared to focal activity (Fig 3A and 3B). This might be attributable to the fact that the wave propagation through the atria due to focal excitation is more uniform and symmetric (around the origin of excitation) than re-entrant excitation. Performing Fourier Transformation analysis (FFT) of the signal from lead V1, commonly used for AF analysis due to its large atrial signal [28] (closest is lead 15 in the 64-lead configuration), allows the fragmentation of the signal V1 to be quantified, providing a way to distinguish the cases of focal from re-entrant excitation waves, with the same excitation rate and origin (Fig 5). FFT MATLAB function was applied to analyze the simulated ECG signals. From the FFT, as would be expected the dominant frequency (DF) shows no marked difference between the focal and re-entrant cases due to the same activation rates. However, the re-entrant cases exhibited considerably more power at higher frequencies. To quantify this, the ratio of the area under the normalized power spectrum density (PSD) in the ranges 0 –(2 x DF) Hz and 0–50 Hz (AFFTr_2DF_) was calculated. The use of the threshold of 2xDF was chosen because it is the value at which the distinction between focal and re-entrant excitation was the most significant (S4 Text). The ratio showed dramatic differences between re-entrant and ectopic activations (Fig 5A, 5B and 5C). By plotting the AFFTr_2DF_ against its DF for all simulations (Fig 5D), it was clear that a ratio of above 0.675 corresponded to focal activity, a ratio below 0.655 corresponded to re-entrant activity and a ratio in the range 0.655 to 0.675 could correspond to either (overlapping area in Fig 5D).

### Algorithm flow chart

**Fig 6:**
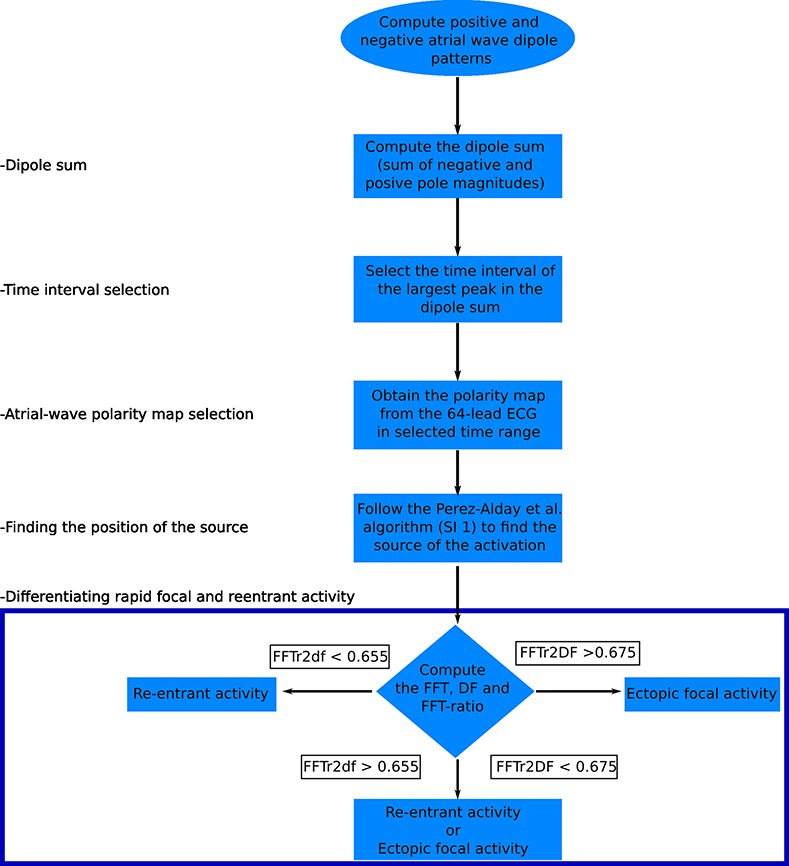
Schematic illustration of the algorithm to identify the location of atrial focal origin or re-entry from atrial wave polarity maps.

The new tools developed were integrated into a flow chart of the algorithm as illustrated in (Fig 6). The first step of the new algorithm was to compute the dipole sum from the body surface potential distribution. Then, by selecting the time interval corresponding to the largest peak in the dipole sum, which is attributable to a large volume of the atrial mass that has been excited, a polarity map can be created. The next step was to implement the previous algorithm we have developed [18] to identify the source of atrial activation based on the body surface potential distribution. The last step was to differentiate focal from re-entrant activities based on the spectral characteristics of the fast atrial waves.

## Results

### Success rate of algorithm

#### Location

The algorithm (Fig 6) was developed based on simulated data of re-entrant excitation waves and ectopic focal activities with their origins located at 10 different sites across the atria. The algorithm was then tested with 20 further simulations to determine its success rate (i.e. the proportion of cases in which the algorithm correctly identified the origin of atrial arrhythmias).

In the test for re-entrant excitation, the success rate of determining the atrial quadrant containing the tip of the scroll wave was 75%. In the cases which the origin of the scroll wave was not identified by the algorithm, it was due to the tip of the scroll wave being close to the boundary of two nearby quadrants (i.e., within 0.5 cm). The direction of the rotation of the scroll wave played an important role as well.

In the test for ectopic focal excitation the success rate for detecting the atrial quadrant where the rapid focal activity was located was 92%. This is comparable with the success rate of our previous algorithm [18] for slow ectopic foci (93%). The consistency between the present and the previous algorithm suggests the newly devised tool for identifying the polarity of the fast atrial wave is valid.

Further refinements to the spatial resolution of the quadrants was performed with the aim to improve the specificity of the algorithm for locating the focal origin site, by dividing each quadrant into sub-quadrants, as described in [18,19]. Though such a spatial refinement improved the detection accuracy in terms of the spatial resolution, the success rate of detection showed a slight decrease, down to 87% and 72% in ectopic and re-entrant activation respectively.

#### Focal *vs* Re-entry

The success rate to differentiate ectopic activity from re-entrant activation with the same frequency was 88%. Note that the algorithm never produced a false positive, because in the remaining 12% of the cases the FFTAr was within the overlapping area where ectopic and re-entrant activity could not be distinguished (Fig 5D, magenta shaded area).

#### White noise added test

The algorithm was tested with random noise added to ECG signals. During this test, a dipole sum was obtained with the same characteristics as previously described. The AFFTr_2DF_ values were affected by the addition of noise; however, the changes did not produce large differences. Further information can be found in S5 Text.

#### Determining the time-dependent location of meandering re-entry

**Fig 7:**
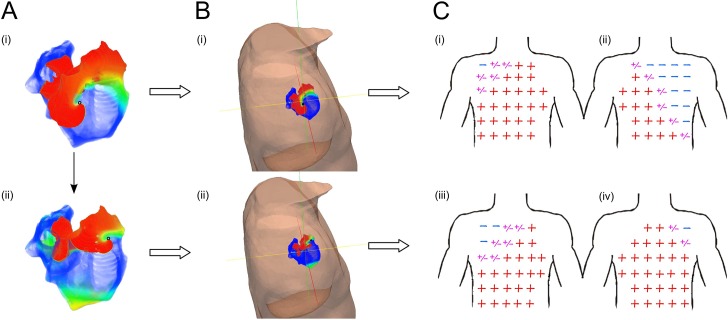
Comparison of quadrants position and atrial-waves polarity maps in meandering re-entrant activation. (A) Snapshot at two different instant of re-entrant activation in the atria. (B) Snapshot at two different instant of re-entrant activation of the atria-torso model from a posterior superior right view. (C) Simulated 64-lead ECG atrial-waves polarity map at two different instant of re-entrant activation. We observed the polarity pattern of the atrial waves of the experimental and simulation, in the anterior (i), (iii) and posterior (ii), (iv) part of the body. The red positive sign represents a positive atrial-wave, the blue negative sign represents negative atrial-wave, and the purple positive/negative sign represents a biphasic atrial-wave.

The algorithm showed good feasibility for tracking the tip of scroll waves with a significant degree of meander. This was done by selecting the time intervals when the tip of the rotor was in two different positions across the atria (Fig 7).

## Discussion

### Major contribution

By using a biophysically detailed computer model of human atria-torso and identifying the correct polarity of the fast atrial waves, we have developed a novel algorithm to locate the origin of atrial fibrillation in association with both of ectopic focal and re-entrant activity. The success rate of the algorithm was 92% and 75% for focal and re-entry activation, respectively. The properties of the FFT allowed re-entry and focal activation to be distinguished with a success rate of 88%.

### Further test

The algorithm was also tested with a different torso geometry. During this test, dipole sum and AFFTr_2DF_ values were obtained and the algorithm successfully identified the quadrant where the origin of the arrhythmia was located. Further information can be found in supporting information S1 Text.

It was also shown that when white noise was added with a signal to noise ratio of 10, the performance of the algorithm was not affected as the changes of the FFTAr values were small (S5 Text). However, further test need to be implemented.

FFT analysis was applied to experimental AF signals, where AFFTr_2DF_ values were obtained to compare simulated and experimental data. Similar AFFTr_2DF_ values were obtained with simulated and experimental data. Further information can be found in S6 Text.

### Comparison to previous/other algorithms

Previous studies have been focused on differentiating ectopic activity against re-entry [29–32]. Most use atria-electrocardiograms to detect and characterize complex fractionated signals, FFT and DF atria maps [29,30,33]. The success rate of these algorithms is in the range of 60–80% [29,30,34], however, as it is an invasive method, it might unduly lengthen the ablation procedure [35]. By using a 12-lead ECG system, algorithms to detect ectopic activity have been developed [16,36], however, the success rates range within 55–78% [15,16,18] and it has been proved that the 12-lead ECG system does not produce enough information to identify the origins when fast atrial waves are presented or under re-entrant activity [15,18,32]. Other attempts have used multi-lead ECG systems and body surface mapping [31,32,37], to correlate to atrial DF or add extra information like phase mapping [32]. However, it has been difficult to validate the time interval, location and the source of the atrial activation when fast atrial waves are presented. Nevertheless, they are promising methods that can add extra useful information. Ours is the first attempt to distinguish the main activity and find the position of the focus and tip of the re-entry from a multi-lead ECG.

The present algorithm can also be used together with other invasive or non-invasive mapping methods, with potential to reduce procedures times in locating the origin of atrial arrhythmias.

### Limitations

The torso model lacks considerations of some other tissue types or organs (such as muscles, fat tissue, bowel, kidneys and spleen) that may affect the amplitude of simulated surface potentials. However, the absence of those tissues does not have a big effect on the polarity of the atrial-waves, which is the characteristic used in the present algorithm, as demonstrated previously [18].

In the algorithm, two-sets of quadrants were defined to cover the torso. The spatial resolution of the 8 quadrant was about 40mm. The finer spatial resolution with lower accuracy was 20mm. Though this provided roughly the site of the AF origin, it is the information needed for identifying which part of the atria for ablation (i.e., left or right atrium, the low or upper part of the atrium). The diameter size in current ablation procedures varies from 3mm to 10mm [9,13,38,39]. Therefore, the current algorithm can be used in clinical studies, but it may require further investigation and refinement to provide the essential information in all case.

### Future work

In the present study, we only tested the effectiveness of the algorithm for detecting single atrial focal activity and a single centre of a rotor activity. However, a possible extension is to identify multiple wavelets, using the dipole evolution patterns. For that purpose, consideration of combined use of the present algorithm with vecto-cardiograms, phase relationships, correlation analysis and inverse problem reconstruction may be necessary, warranting further investigation.

## Conclusion

A novel algorithm has been developed to locate the origins of rapid and irregular atrial excitation waves, associated with both ectopic focal and re-entrant activity. This represents a significant progress to previously developed algorithms to predict AF origins in association with focal activities.

## Supporting information

S1 Table Table of conductivities. (DOCX)

S1 Text Female Torso. (DOCX)

S2 Text Atrial Model. (DOCX)

S3 Text Colman_2013_single_cell_function. (C)

S1 Fig 2D and 3D view files. (ZIP)

S2 Fig Dipole Validation. (DOCX)

S3 Fig Perez-Alday et al. algorithm. (DOCX)

S4 Text FFTR ratio. (DOCX)

S5 Text White Noise. (DOCX)

S6 Text Experimental Data. (DOCX)
