# Supplementary material for: Novel non-invasive algorithm to identify the origins of re-entry and ectopic foci in the atria from 64-lead ECGs: A computational study
Source: PLoS Comput Biol. 2017 Mar 2;13(3):e1005270. doi: 10.1371/journal.pcbi.1005270 (PMC5333795; doi:10.1371/journal.pcbi.1005270)
Supplement: S1 Table — (DOCX) [file pcbi.1005270.s001.docx]

**Suporting information Table S1**

Novel non-invasive algorithm to identify the origins of re-entry and ectopic foci in the atria from 64-lead ECGs. A computational study.

Erick A. Perez Alday^1^, Michael A. Colman^2^, Philip Langley ^3^, Henggui Zhang^1*^

*^1^ Biological Physics Group, Department of Physics and Astronomy, University of Manchester, Manchester, United Kingdom,*

*^2^Theoretical Physics Division, Department of Physics and Astronomy, University of Manchester, Manchester, United Kingdom*

*^3^School of Engineering, University of Hull, Hull, United Kingdom,*

*^*^Correspondence: henggui.zhang@manchester.ac.uk*

Table S1. Tissue conductivity.

| **Tissue Type** | **Conductivity (Sm^-1^)** |
| --- | --- |
| Thorax | 0.2 |
| Lungs | 0.08 |
| Liver | 0.15 |
| Stomach | 0.12 |
| Kidneys | 0.07 |
| Bone | 0.005 |
| Blood | 0.6 |
| Fat | 0.05 |
| Myocardium | 0.25 |

The conductivities for the individual tissues were based on values derived from the literature, and can be found in (1–5).

1. Jolley M, Stinstra J, Pieper S, MacLeod R, Brooks DH, Cecchin F, et al. A computer modeling tool for comparing novel ICD electrode orientations in children and adults. Heart Rhythm. 2008 Apr;5(4):565–72.

2. Geddes LA, Baker LE. The specific resistance of biological material—A compendium of data for the biomedical engineer and physiologist. Med Biol Eng. 1967 May;5(3):271–93.

3. Grimnes S, Martinsen ØG. Chapter 4 - PASSIVE TISSUE ELECTRICAL PROPERTIES. In: Bioimpedance and Bioelectricity Basics (Second Edition). New York: Academic Press; 2008. p. 93–137.

4. Rush S, Abildskov JA, Mcfee R. Resistivity of Body Tissues at Low Frequencies. Circ Res. 1963 Jan 1;12(1):40–50.

5. Gabriel C, Gabriel S, Corthout E. The dielectric properties of biological tissues. Phys Med Biol. 1996 Nov;41(11):2231–49.
