# Supplementary material for: Novel non-invasive algorithm to identify the origins of re-entry and ectopic foci in the atria from 64-lead ECGs: A computational study
Source: PLoS Comput Biol. 2017 Mar 2;13(3):e1005270. doi: 10.1371/journal.pcbi.1005270 (PMC5333795; doi:10.1371/journal.pcbi.1005270)
Supplement: S1 Text — (DOCX) [file pcbi.1005270.s002.docx]

**Supporting information S1 Text.**

Novel non-invasive algorithm to identify the origins of re-entry and ectopic foci in the atria from 64-lead ECGs. A computational study.

Erick A. Perez Alday^1^, Michael A. Colman^2^, Philip Langley ^3^, Henggui Zhang^1*^

*^1^ Biological Physics Group, Department of Physics and Astronomy, University of Manchester, Manchester, United Kingdom,*

*^2^Theoretical Physics Division, Department of Physics and Astronomy, University of Manchester, Manchester, United Kingdom*

*^3^School of Engineering, University of Hull, Hull, United Kingdom,*

*^*^Correspondence: henggui.zhang@manchester.ac.uk*

Re-entrant and ectopic focus activation with origins in the inferior vena cava (IVC) (Fig A) were tested within a female torso model. The position of the atria can be observed in Fig A- Aii-iii. AFFTr_2DF_ values of Lead-15 were obtained to differentiate ectopic and re-entrant activation (Fig A-B). Subsequently, a dipole sum was calculated and the time interval of the main activation in each case was selected (Fig A-C). Polarity maps were then obtained in each case (Fig A-D). The algorithm correctly identifies the correct quadrant in each case.


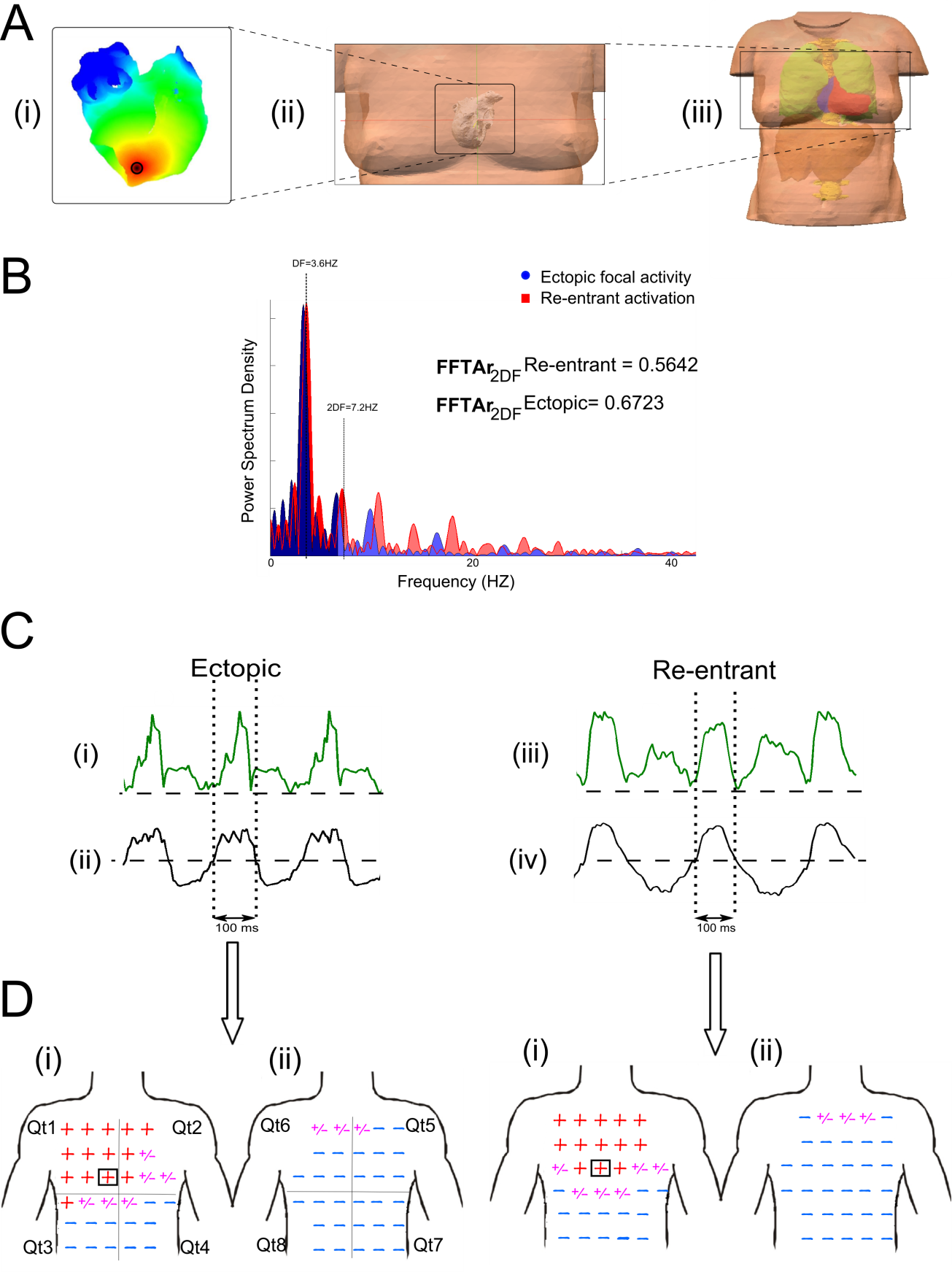


**Fig A. Illustration of algorithm implementation in a torso woman.** (A): Illustration of atria ectopic activation focus in the inferior vena cava (i) placed inside a female torso model (ii). (B) Power spectral density for ectopic focal (blue) and re-entrant (red) activity located in the IVC. The darker shadow corresponds to the area between 0 – 2 x Dominant frequency (DF). AFFTr_2DF_ is the ratio of the area under the power spectrum density in the ranges 0 – (2 x DF) Hz and (2 x DF) – 50 Hz: AFFTr_2DF_ = Area_0-2DF_/Area_0-50Hz_. (C): Dipole sum (green line) (i) and lead V1 (black line) (ii), (i) were used to identify the time interval (section between dotted lines) of ectopic or re-entrant patterns where the tip was located in IVC. (D): Atrial-wave polarity map in the anterior (i) and posterior (ii) part of the torso for atrial activation initiated at IVC. A red sign represents a positive polarity in the atrial-wave, the blue sign is a negative polarity and a purple sign represents a biphasic atrial-wave. The black square represents the electrode position of lead V1. The anterior (ii) and posterior (i) parts of the torso are shown for each case. In each case the algorithm correctly identifies the correct quadrant.
