# Supplementary material for: Novel non-invasive algorithm to identify the origins of re-entry and ectopic foci in the atria from 64-lead ECGs: A computational study
Source: PLoS Comput Biol. 2017 Mar 2;13(3):e1005270. doi: 10.1371/journal.pcbi.1005270 (PMC5333795; doi:10.1371/journal.pcbi.1005270)
Supplement: S2 Text — (DOCX) [file pcbi.1005270.s003.docx]

Supplementary Material Text S2

**A new algorithm to diagnose atrial ectopic origin from multi lead ECG systems – insights from 3D virtual human atria and torso**

Erick A. Perez Alday^1*^, Michael A. Colman^1*^, Philip Langley^2^, Timothy D. Butters^1^, Jonathan Higham^1^, Antony J. Workman^3^, Jules C. Hancox^1 4^, Henggui Zhang^1+^

*^1^ Biological Physics Group, Department of Physics and Astronomy, University of Manchester, Manchester, United Kingdom,*

*^2^School of Engineering, University of Hull, Hull, United Kingdom,*

*^3^Institute of Cardiovascular and Medical Sciences, University of Glasgow, Glasgow, United Kingdom,*

*^4^School of Physiology, Pharmacology and Cardiovascular Research Laboratories, School of Medical Sciences, University of Bristol, Bristol, United Kingdom.*

^*^*Both authors have contributed equally to this study.*

*^+^Correspondence: henggui.zhang@manchester.ac.uk*

Most of the main features of the Models and the key simulation steps has been proper referred or included in the main paper. However, we have described the key features of the models in this Supplementary Material Text S1..

**Atrial model**

The Colman et al. family[21,22]–[1] of single cell models, which accounts for the action potential in all of the major heterogeneous regions of the atria, is utilised in this study. The model is based on the Courtemanche-Ramirez-Nattel model, updated to account for recent experimental data and a compartmentalised intracellular calcium handling model [2]. Data regarding regional differences in ion current density and kinetics from both human and canine were used to derive the family of cell models, using the right-atrial cell model as a base. For further details of single cell model development and validation the reader is referred to [22].

The 3D anatomical model is extracted from the visible human female dataset and includes rule-based fibre orientation along the preferential conduction pathways of the CT, PM and BB [3]. The model was then integrated with a realistic anatomical model of the human sinus node [21], [4] and further segmented to account for the major regions of the atria, including the right and left atrial appendages, pulmonary veins, atrial-septum and atrio-ventricular ring [22].

The electrical activity in cardiac tissue is described by the reaction-diffusion equation [5]:

$$\frac{\partial V_{m}}{\partial t}=\nabla\cdot\left( \boldsymbol{D}\nabla V_{m} \right)-\frac{I_{ion}}{C_{m}}$$

Where $V_{m}$ is the membrane potential, ***D*** is the tensor of diffusion coefficients describing the rate of conduction via gap junctional currents, $I_{ion}$ is the total ionic current in a single cell and $C_{m}$ is the membrane capciatnce. The Forward-Euler method is used to integrate the differential equations in the single cell model. A finite-difference method is used to solve the reaction-diffusion equation describing coupling of cells in 3D. Lookup tables are also implemented to improve computational efficiency. Furthermore, initial conditions are read in dynamically based on a 1D strand model and the relevant model parameters (e.g. region, BCL) to speed convergence of the model in 3D. Such an approach allows fewer conditioning stimuli to be used in 3D, significantly improving computation time.

In tissue modelling, AF remodelling was induced using the data and model parameters of Table A [22].

| Parameters |  | AF |
| --- | --- | --- |
| I_CaL_ |  | -70% |
| I_Kur_ |  | -50% |
| I_to_ |  | -65% |
| I_K1_ |  | +100% |
| I_Ks_ |  | +100% |
| I_NaCa_ |  | +55% |
| I_Kr_ |  | No change |
| SERCA |  | +50% |
| RyR |  | +300% |
| SR Ca^2+^ leak |  | +25% |

Supporting References

[1] M. A. Colman, M. Varela, J. C. Hancox, H. Zhang, and O. V. Aslanidi, ‘Evolution and pharmacological modulation of the arrhythmogenic wave dynamics in canine pulmonary vein model’, *Eur. Eur. Pacing Arrhythm. Card. Electrophysiol. J. Work. Groups Card. Pacing Arrhythm. Card. Cell. Electrophysiol. Eur. Soc. Cardiol.*, vol. 16, no. 3, pp. 416–423, Mar. 2014.

[2] J. T. Koivumäki, T. Korhonen, and P. Tavi, ‘Impact of Sarcoplasmic Reticulum Calcium Release on Calcium Dynamics and Action Potential Morphology in Human Atrial Myocytes: A Computational Study’, *PLoS Comput Biol*, vol. 7, no. 1, p. e1001067, Jan. 2011.

[3] G. Seemann, C. Höper, F. B. Sachse, O. Dössel, A. V. Holden, and H. Zhang, ‘Heterogeneous three-dimensional anatomical and electrophysiological model of human atria’, *Philos. Transact. A Math. Phys. Eng. Sci.*, vol. 364, no. 1843, pp. 1465–1481, Jun. 2006.

[4] N. Chandler, O. Aslanidi, D. Buckley, S. Inada, S. Birchall, A. Atkinson, D. Kirk, O. Monfredi, P. Molenaar, R. Anderson, V. Sharma, D. Sigg, H. Zhang, M. Boyett, and H. Dobrzynski, ‘Computer three-dimensional anatomical reconstruction of the human sinus node and a novel paranodal area’, *Anat. Rec. Hoboken NJ 2007*, vol. 294, no. 6, pp. 970–979, Jun. 2011.

[5] R. H. Clayton, O. Bernus, E. M. Cherry, H. Dierckx, F. H. Fenton, L. Mirabella, A. V. Panfilov, F. B. Sachse, G. Seemann, and H. Zhang, ‘Models of cardiac tissue electrophysiology: progress, challenges and open questions’, *Prog. Biophys. Mol. Biol.*, vol. 104, no. 1, pp. 22–48, 2011.
