# Supplementary material for: Novel non-invasive algorithm to identify the origins of re-entry and ectopic foci in the atria from 64-lead ECGs: A computational study
Source: PLoS Comput Biol. 2017 Mar 2;13(3):e1005270. doi: 10.1371/journal.pcbi.1005270 (PMC5333795; doi:10.1371/journal.pcbi.1005270)
Supplement: S2 Fig — (DOCX) [file pcbi.1005270.s006.docx]

**Supporting Information S2 Fig.**

Novel non-invasive algorithm to identify the origins of re-entry and ectopic foci in the atria from 64-lead ECGs. A computational study.

Erick A. Perez Alday^1^, Michael A. Colman^2^, Philip Langley ^3^, Henggui Zhang^1*^

*^1^ Biological Physics Group, Department of Physics and Astronomy, University of Manchester, Manchester, United Kingdom,*

*^2^Theoretical Physics Division, Department of Physics and Astronomy, University of Manchester, Manchester, United Kingdom*

*^3^School of Engineering, University of Hull, Hull, United Kingdom,*

*^*^Correspondence: henggui.zhang@manchester.ac.uk*

**
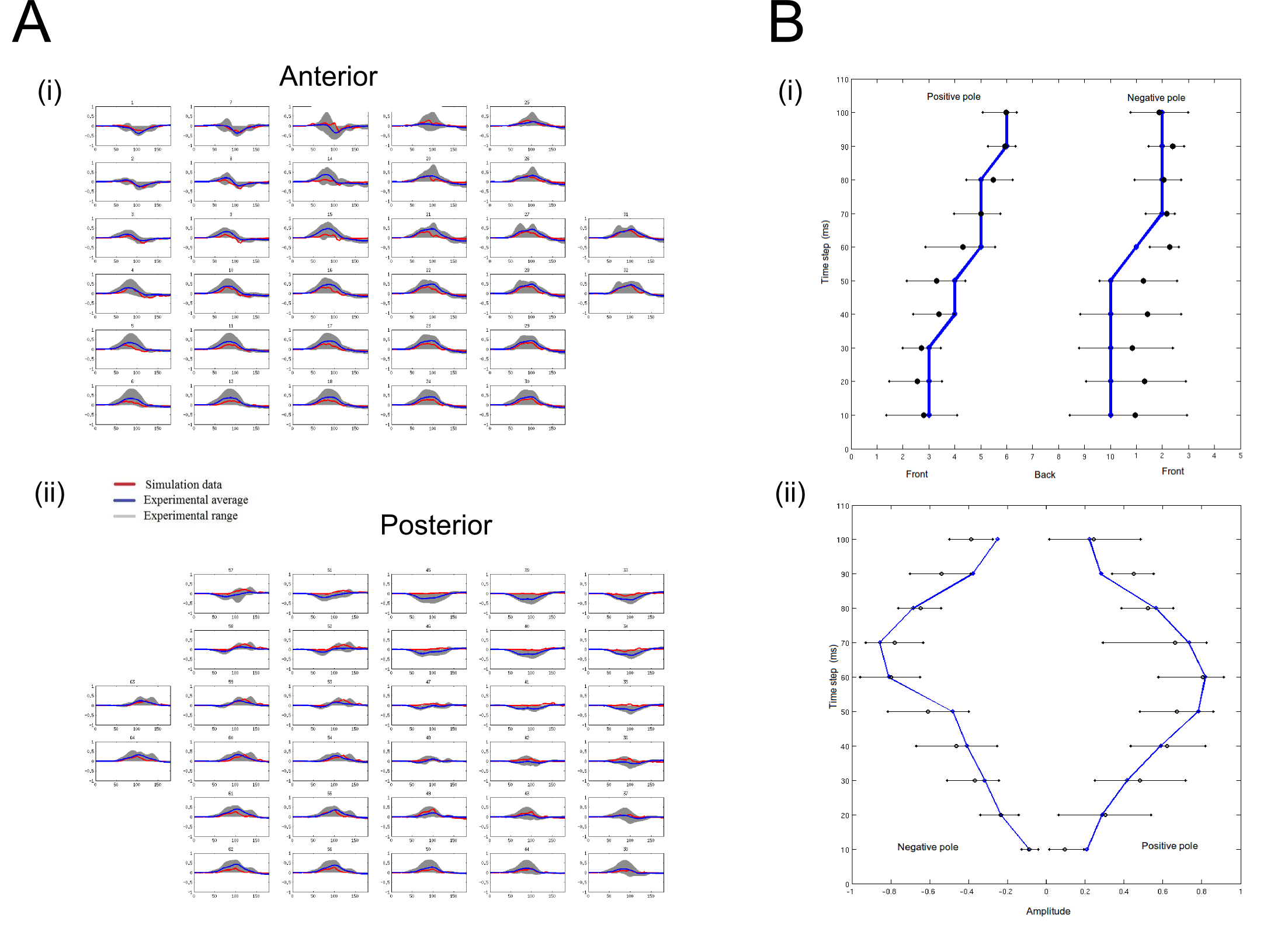
**

**S2 Fig. Comparison of p-waves and dipole evolution between the simulated and experimental data**. A and B Comparison of the simulated 64-lead ECG P-waves polarity (ii) to experimental data (i). In this Fig, the arrangement of the P-waves is set out to match electrode placement (see Fig 1). We observed the polarity pattern of the P-waves of the experimental and simulation, in the front (A) and back (B) part of the body. The red positive sign signifies an upright P-wave, the blue negative sign represents an inverted P-wave, and the purple positive/negative sign represents a biphasic P-wave. C Spatial (i) and amplitude (ii) temporal evolution of the dipole. The black dots and lines are the experimental data and error bar taken from [27], and the blue lines and dots are obtained from our simulation during a stimuli applied to the superior part of the sino-atrial node region. In (i) the horizontal axis is a continuous scale from the first vertical line electrodes (1-6) to the last line of electrodes (33-38), without taking in to account 31, 32, 63 and 64. Fig adapted from [18,27]
