# Supplementary material for: Novel non-invasive algorithm to identify the origins of re-entry and ectopic foci in the atria from 64-lead ECGs: A computational study
Source: PLoS Comput Biol. 2017 Mar 2;13(3):e1005270. doi: 10.1371/journal.pcbi.1005270 (PMC5333795; doi:10.1371/journal.pcbi.1005270)
Supplement: S3 Fig — (DOCX) [file pcbi.1005270.s007.docx]

**Suporting information Figure S3**

Novel non-invasive algorithm to identify the origins of re-entry and ectopic foci in the atria from 64-lead ECGs. A computational study.

Erick A. Perez Alday^1^, Michael A. Colman^2^, Philip Langley ^3^, Henggui Zhang^1*^

*^1^ Biological Physics Group, Department of Physics and Astronomy, University of Manchester, Manchester, United Kingdom,*

*^2^Theoretical Physics Division, Department of Physics and Astronomy, University of Manchester, Manchester, United Kingdom*

*^3^School of Engineering, University of Hull, Hull, United Kingdom,*

*^*^Correspondence: henggui.zhang@manchester.ac.uk*

**S3 Fig. Schematic illustration of the algorithm to identify the quadrant of atrial focal origin based on 64-lead ECG P-wave values. Fig adapted from [18].**


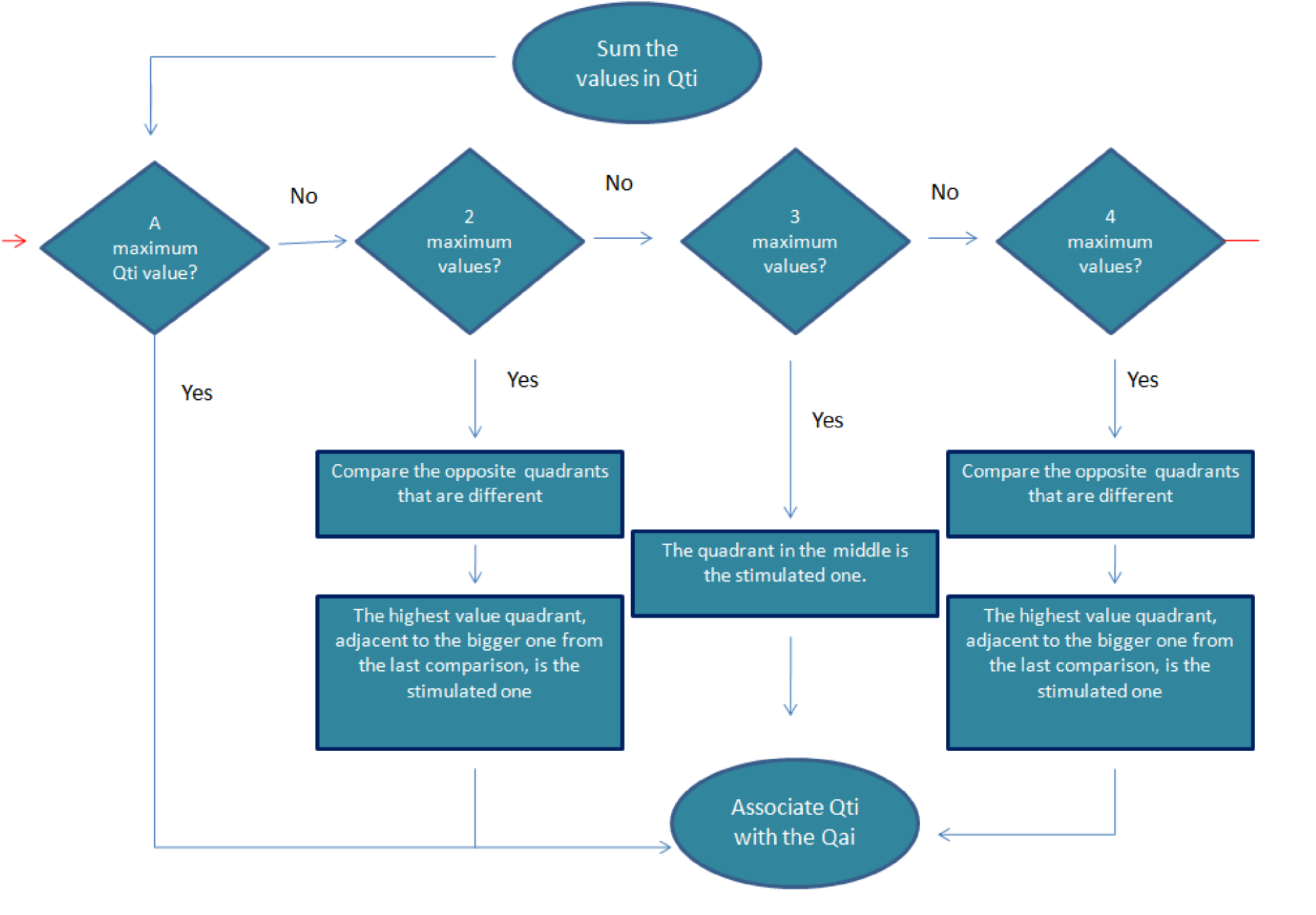


**S3 Fig. Schematic illustration of the algorithm to identify the quadrant of atrial focal origin based on 64-lead ECG P-wave values. Fig adapted from** [18**].**
