# Supplementary material for: Novel non-invasive algorithm to identify the origins of re-entry and ectopic foci in the atria from 64-lead ECGs: A computational study
Source: PLoS Comput Biol. 2017 Mar 2;13(3):e1005270. doi: 10.1371/journal.pcbi.1005270 (PMC5333795; doi:10.1371/journal.pcbi.1005270)
Supplement: S4 Text — (DOCX) [file pcbi.1005270.s008.docx]

**Supporting information S4 Text.**

Novel non-invasive algorithm to identify the origins of re-entry and ectopic foci in the atria from 64-lead ECGs. A computational study.

Erick A. Perez Alday^1^, Michael A. Colman^2^, Philip Langley ^3^, Henggui Zhang^1*^

*^1^ Biological Physics Group, Department of Physics and Astronomy, University of Manchester, Manchester, United Kingdom,*

*^2^Theoretical Physics Division, Department of Physics and Astronomy, University of Manchester, Manchester, United Kingdom*

*^3^School of Engineering, University of Hull, Hull, United Kingdom,*

*^*^Correspondence: henggui.zhang@manchester.ac.uk*

The overlapping area, region where re-entrant and ectopic activation cannot be distinguished (magenta regions in Fig A: 1DF, 2DF, 3DF), depends on the frequency used to calculate the AFFTr ratio, this frequency depends on the dominant frequency (DF) of each activation.

$$\mathrm{AFFTr}_{1DF}=\frac{Area under the FFT curve between 0 and the DF in Hz.}{Area under the FFT curve between 0 and 50 Hz.}$$

$$\mathrm{AFFTr}_{2DF}=\frac{Area under the FFT curve between 0 and 2 times the DF in Hz.}{Area under the FFT curve between 0 and 50 Hz.}$$

$$\mathrm{AFFTr}_{3DF}=\frac{Area under the FFT curve between 0 and 3 times the DF in Hz.}{Area under the FFT curve between 0 and 50 Hz.}$$

Once the ratios are calculated, scatter plots of the ratio values against the DF are created to find the ratio at which the overlapping area is minimized.


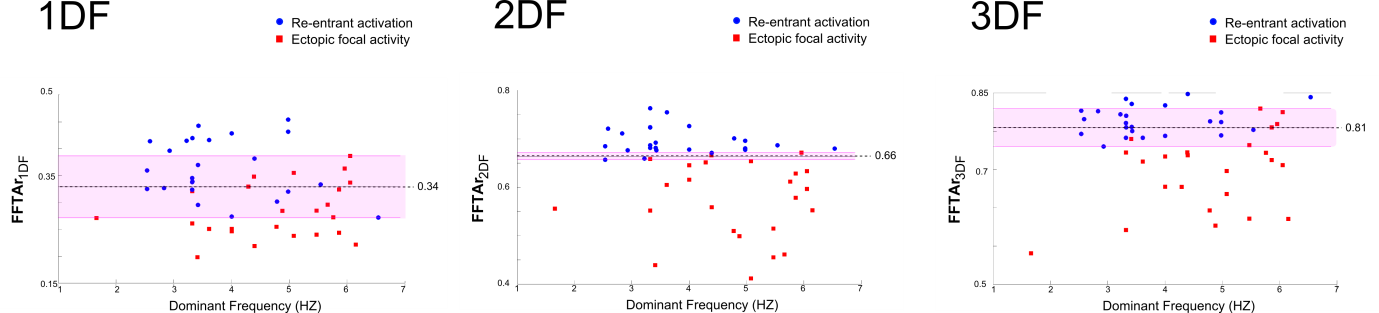


**Fig A.** Scatter plots of different (AFFTr) against the DF, the magenta area is the overlapping area where both activities can occur. 1DF is (AFFTr_1DF_) against the DF. 2DF is (AFFTr_2DF_) against the DF. 3DF is (AFFTr_3DF_) against the DF.

The properties of the scatter plots of different (AFFTr) against DF are displayed in Fig B. Fig B-A, shows the width of the overlapping area against the DF. Fig B-B, shows the number of cases (re-entrant and focal ectopic activation) inside the overlapping area against the DF. Fig B-C, shows the number of focal ectopic activation inside the overlapping area against the DF. Fig B-D, shows the number of re-entrant activation in the overlapping area against the DF.


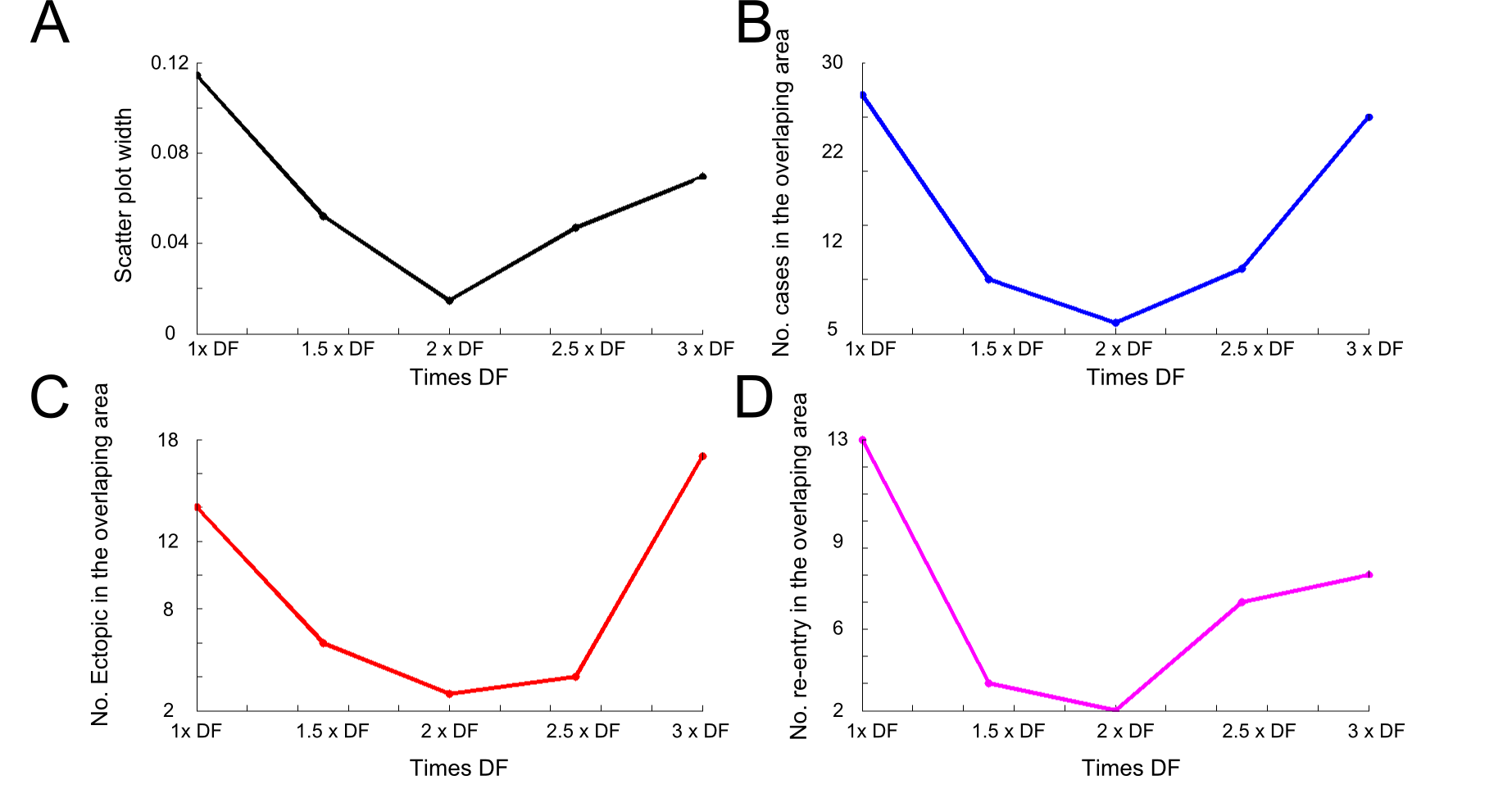


**Fig B.** Properties of the overlapping area from scatter plots against different values of the dominant frequency (DF). (A) Width of the overlapping area from scatter plot vs values of DF. (B) All simulation cases (Ectopic focal and re-entrant activity) that are inside the overlapping area vs values of DF. (C) Number of Ectopic focal activity simulations inside the overlapping area vs values of DF. (D) Number of Re-entrant activity simulations inside the overlapping area vs values of DF.
