# Supplementary material for: Novel non-invasive algorithm to identify the origins of re-entry and ectopic foci in the atria from 64-lead ECGs: A computational study
Source: PLoS Comput Biol. 2017 Mar 2;13(3):e1005270. doi: 10.1371/journal.pcbi.1005270 (PMC5333795; doi:10.1371/journal.pcbi.1005270)
Supplement: S5 Text — (DOCX) [file pcbi.1005270.s009.docx]

**Supporting information Text S5.**

Novel non-invasive algorithm to identify the origins of re-entry and ectopic foci in the atria from 64-lead ECGs. A computational study.

Erick A. Perez Alday^1^, Michael A. Colman^2^, Philip Langley ^3^, Henggui Zhang^1*^

*^1^ Biological Physics Group, Department of Physics and Astronomy, University of Manchester, Manchester, United Kingdom,*

*^2^Theoretical Physics Division, Department of Physics and Astronomy, University of Manchester, Manchester, United Kingdom*

*^3^School of Engineering, University of Hull, Hull, United Kingdom,*

*^*^Correspondence: henggui.zhang@manchester.ac.uk*

White noise was added to re-entrant and ectopic focus activations with origins in the right atria appendage (RAA) to test the performance of the algorithm under noise situation. The dipole sum was calculated in order to select the time interval of the main activation, and then, the FFTAr_2DF_ ratio of Lead-15 was computed to differentiate ectopic and re-entrant activation.

20% and 40% white noise (σ) was added to the simulated signal using the formula:

$$\sigma=Rnd*RMS*f$$

Where *Rnd* are random numbers, *RMS* is the root mean square value of the body surface potentials over the entire time period to be analyzed, and *f* is the percentage of noise to be added, i.e. *f*=.2 for 20% noise level, a signal to noise ratio (SNR) of 10 was used in all cases.


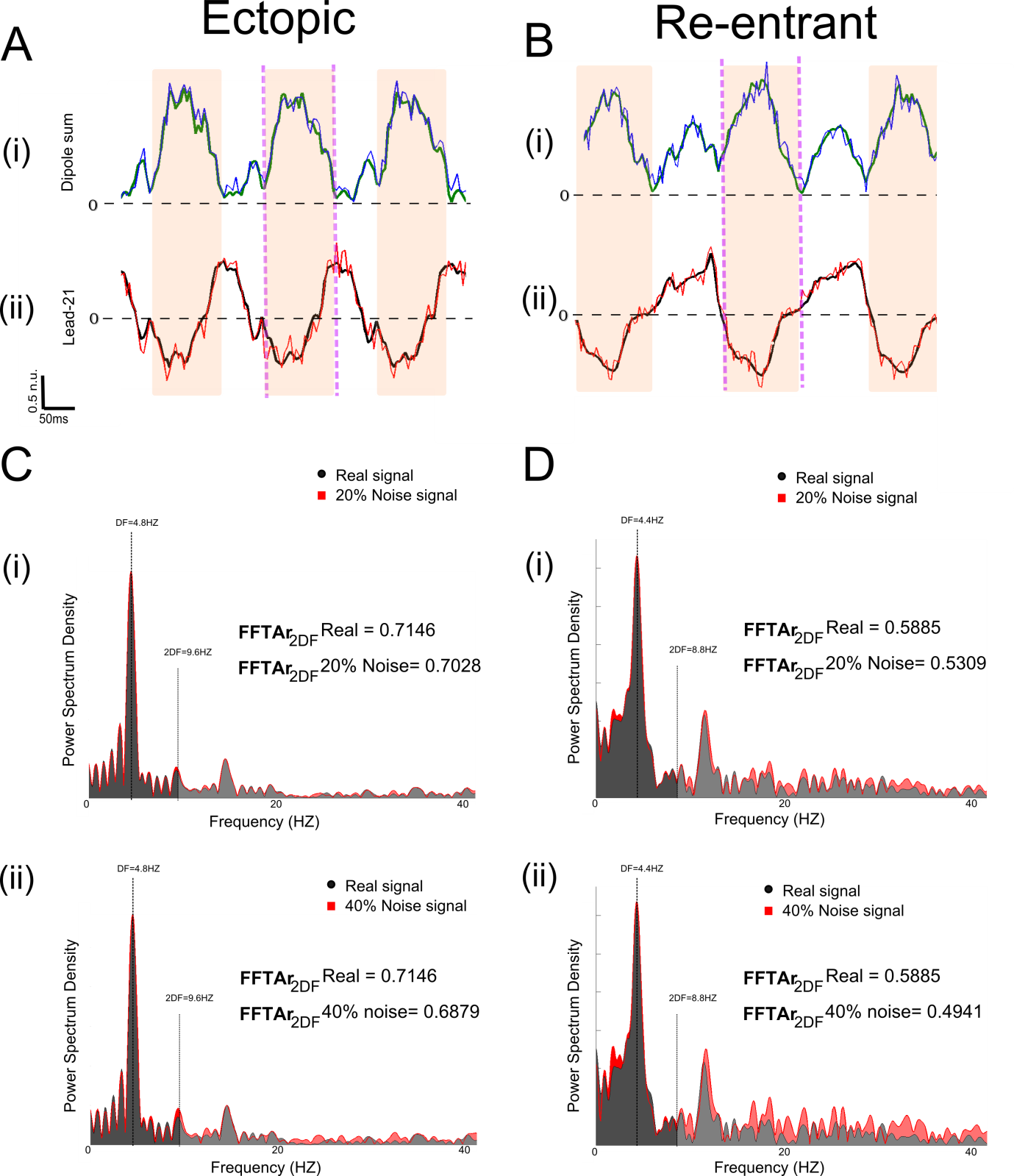


**Fig A. Illustration of dipole sum and FFT of ectopic and re-entrant activation focus in right atrial appendage with added white noise.** Dipole sum (green line) with added white noise (blue line) (i) and lead 21 (black line) with added white noise (red line) (ii), were used to identify the time interval (section in magenta shadow) of ectopic (A) or re-entrant (B) patterns where the tip was located in right atrial appendage (RAA). Power spectral density for ectopic focal (C) and re-entrant (D) activity without noise (gray shadow) and with added noise (red shadow) at 20% (C-i and D-i) and 40% (C-ii and D-ii) noise level, located in the RAA. The darker shadow corresponds to the area between 0 – 2 x Dominant frequency (DF). AFFTr_2DF_ is the ratio of the area under the power spectrum density in the ranges 0 – (2 x DF) Hz and (2 x DF) – 50 Hz: AFFTr_2DF_ = Area_0-2DF_/Area_0-50Hz_.
