# Supplementary material for: Novel non-invasive algorithm to identify the origins of re-entry and ectopic foci in the atria from 64-lead ECGs: A computational study
Source: PLoS Comput Biol. 2017 Mar 2;13(3):e1005270. doi: 10.1371/journal.pcbi.1005270 (PMC5333795; doi:10.1371/journal.pcbi.1005270)
Supplement: S6 Text — (DOCX) [file pcbi.1005270.s010.docx]

**Supporting information Text S6.**

Novel non-invasive algorithm to identify the origins of re-entry and ectopic foci in the atria from 64-lead ECGs. A computational study.

Erick A. Perez Alday^1^, Michael A. Colman^2^, Philip Langley ^3^, Henggui Zhang^1*^

*^1^ Biological Physics Group, Department of Physics and Astronomy, University of Manchester, Manchester, United Kingdom,*

*^2^Theoretical Physics Division, Department of Physics and Astronomy, University of Manchester, Manchester, United Kingdom*

*^3^School of Engineering, University of Hull, Hull, United Kingdom,*

*^*^Correspondence: henggui.zhang@manchester.ac.uk*

Fast Fourier Transformation analysis (FFT) MATLAB function was applied to different experimental lead V1 signals (Fig A - A). The sample rate was 500Hz. The sample time was 500 ms. The analysis was made over the first 50Hz of the power spectrum density (PSD) signals. PSD signals were normalized for comparison proposes. AFFTr_2DF_ values were obtained for four different experimental data were F-waves were presented (Fig A- B). The PSD signals. It can be observed how the AFFTr_2DF_ values decreased with more fragmented ECG signals (Fig A – B i-iv).


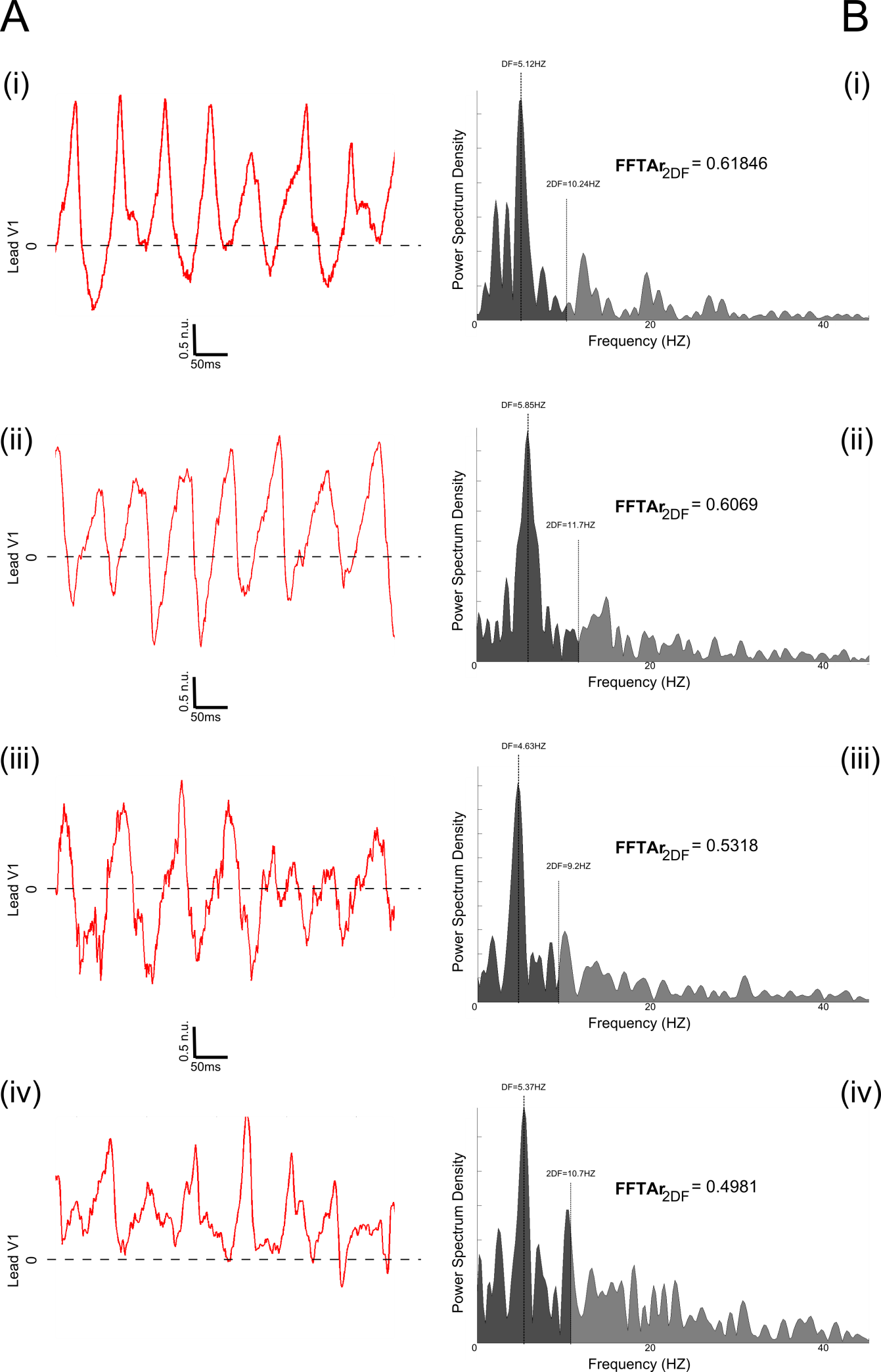


**Fig A**. Illustration of AFFTr_2DF_ obtained for different experimental Lead v1 signals with F-waves**.** (A): Lead V1 signals with F-waves presented. (B) Power spectral density of each ECG signal. The darker shadow corresponds to the area between 0 – 2 x Dominant frequency (DF). AFFTr_2DF_ is the ratio of the area under the power spectrum density in the ranges 0 – (2 x DF) Hz and (2 x DF) – 50 Hz: AFFTr_2DF_ = Area_0-2DF_/Area_0-50Hz_.
